# Comparative Hepatoprotective Effect of Vitamins A and E Against Gasoline Vapor Toxicity in Male and Female Rats

**DOI:** 10.4021/gr2009.10.1318

**Published:** 2009-09-20

**Authors:** Friday Effiong Uboh, Patrick E. Ebong, Ime B. Umoh

**Affiliations:** a Department of Biochemistry, Faculty of Basic Medical Science, College of Medical Sciences, University of Calabar, Calabar, Nigeria

**Keywords:** Hepatoprotective, Gasoline vapors, Retinol, Alpha-tocopherol, Aminotransferases, Bilirubin

## Abstract

**Background:**

Plasma alanine transferase(ALT), aspartate transferase(AST), α-glutamyl transferase(GGT), and alkaline phosphatase(ALP) activities are known biomarkers in assessing hepatic functional integrity. A remarkable rise in the activities of these enzymes normally signifies hepatotoxicity of chemical agent(s) in the biological system. Exposure to 17.8 cm^3^h^-1^m^-3^ of PMS blend unleaded gasoline vapors (UGV) for 6 hr/day, 5 days/week for 20 weeks have been reported to cause hepatotoxicity in rats.

**Methods:**

In this study, the comparative hepatoprotective effect of vitamins A (retinol) and E (α-tocopherol) against UGV-induced toxicity was assessed in male and female rats. Retinol and α-tocopherol at prophylactic dosage (400 and 200 IU/kg/day, respectively) were separately administered orally to the test rats concomitant with exposure to UGV in the last two weeks of the experiment.

**Results:**

The results of this study indicated that exposure to UGV caused significant increase (P < 0.05) in the activities of serum ALT, AST, ALP, GGT and bilirubin in male and female rats. Oral administration of prophylactic doses of retinol and α-tocopherol produced a significant decrease (P < 0.05) in the activities of these parameters in male and female test rats, compared with the non-treated test rats; but insignificant increase(P ≥ 0.05), compared with the control. However, the hepatoprotective effect of α-tocopherol was observed to be more potent than that of retinol.

**Conclusions:**

The result of this study demonstrated that the hepatoprotective potency of α-tocopherol against gasoline vapors toxicity was higher than that of retinol in male and female rats, although the female gender of the animal model responded to treatment with both vitamins better than the males. Hence, the work suggested the beneficial effects of both vitamins against hepatotoxicity in individuals frequently exposed to gasoline vapors.

## Introduction

Gasoline is widely used as fuels for automobiles and some electricity generating machines. It is known a very volatile liquid, with several organic and inorganic constituents. Direct evaporation of liquid gasoline releases gasoline vapors into the immediate surroundings. These vapors are ubiquitous in the environment and constitute various components of petroleum pollutants in the air, which are of great environmental and human health concern. Exposures to these pollutants are common in the refineries, oil fields, refueling stations, petrochemical industries, motor mechanical workshops, and traffic-congested areas. A good percent of human populace is directly or indirectly exposed to these pollutants in the course of their day to day activities. Generally, those occupationally exposed constitute the population at greater risk of frequent exposure [1, 2]. The potential health hazards associated with chronic or sub-chronic exposure to these ubiquitous pollutants in the environment has attracted the attention of the general public and scientific community in particular.

It has been reported that more saturated than unsaturated aromatic hydrocarbons are found in human and animal blood after inhalation exposure to equal concentrations a mixture of C_8-10_ compounds [3]. In animals, exposure to gasoline vapors have been shown to produce various toxicity effect in many tissues. In our previous studies, it was observed and reported that gasoline vapors induced proatherogenic changes in serum lipid profile and signs of hepatic oxidative stress [4-6], haematotoxicity [7, 8], reproductive toxicity [9] and nephrotoxicity [10], in male and female rats. The basic molecular mechanisms through which gasoline vapors’ constituents and other chemical agents express their toxicity effects may vary. For instance, it has been reported that the molecular mechanism that may be responsible for the toxicity of cadmium involve oxidative stress which disturbs the antioxidant defense system and produces reactive oxygen species(ROS), including hydrogen peroxide, superoxide and hydroxyl radical [11]. Our previous studies showed that exposure to gasoline vapors also caused oxidative stress which disturbs the antioxidant defense system and produces alteration in lipid peroxidation [6, 8].

The major concern of the environmental and biochemical toxicologists in the recent times has been how to devise measures that can abate or reverse the adverse effects associated with exposure to ubiquitous environmental pollutants. Since the report of our previous studies suggested that the toxicity effect associated with exposure to gasoline vapors’ constituents may be an indication of tissue, or tissue components - reactive metabolite species interactions in the body; it is believed that the presence of antioxidants may reverse or protect against its toxicity effects. Some antioxidants are naturally present in the body, while others have to be provided as micronutrients in the diets. Some vitamins are known to play an important role in ameliorating the toxicity effects of reactive species generated by chemical agents in the biological systems. Vitamins A and E are among the antioxidants vitamins that have attracted the attention of biochemical and toxicological researchers in the recent times.

Vitamin A, a member of retinoid family, is obtained from β-carotene. It exists in several chemical forms, such as retinol, retinoic acid and retinal. Interconversions between these chemical forms readily occur in the body. Vitamin A is also present as a retinyl ester in the tissues of animals. Retinol and the related compounds are reported to possess apparent ability to interfere with carcinogenesis. Administration of retinol and other retinoids to animals is reported to delay, arrest and even reverse progression of premalignant cells and malignant characteristics. Vitamin E is another important fat-soluble antioxidant vitamin. There are about eight naturally occurring tocopherols with vitamin E activity. Among these, α- tocopherol is considered to be the most important tocopherol, since it has been reported to constitute about 90% of the tocopherols in animal tissues and displays the greatest biological activity in bioassay systems [12]. Among the various biochemical functions of the vitamins A and E, their antioxidative and protective role have attracted more investigations in the recent times [13-17].

Vitamin A is reported to enhance a marked hepatoprotective effect against CCl-induced hepatic damage in mice [18], cadmium toxicity in rats [19], gasoline vapors induced hepatotoxicity in rats [20]. Retinol and the related compounds have been reported to possess apparent ability to interfere with some chemical reactions that may potentiate carcinogenesis [13]. We have also observed and reported that administration of vitamin A and E produced a significant regain in weight loss, growth depression and haematotoxicity resulting from exposure to gasoline vapors in male and female rats [21]. It has been documented that the primary antioxidant role of vitamins A and E is to scavenge singlet oxygen; the singlet oxygen reacts with lipids to form lipid hydroperoxides, and the removal of singlet oxygen prevents lipid peroxidation [22]. Hence, among the various biochemical functions of vitamins A and E, their antioxidative and protective role have attracted more investigations in the recent times [12, 16, 17]. The α-tocopherol is observed to be the major lipid soluble antioxidant vitamin protecting membranes and lipoproteins from injury by free radicals. Since administration of retinol and α-tocopherol to animals has been reported to delay, arrest and even reverse the progression of various chemical toxicities in different animal models, this study was designed to assess the comparative protective potential of vitamins A and E against gasoline vapors-induced hepatotoxicity in male and female rats.

## Materials and Methods

### Experimental animals

Forty-eight matured Wistar albino rats (twenty-four females and twenty-four males) weighing 160.0 ± 20.8 g were obtained from the animal house of the College of Medical Sciences, University of Calabar, Calabar, Nigeria, and used for this study. The rats were divided into six groups with six rats each, as follows:

Group I (Mc): Male control group, without exposure to gasoline vapors.

Group II (Mt): Male test group, exposed to gasoline vapors only.

Group III (MvitA): Male test group concomitantly administered with vitamin A daily

for the last two weeks of the exposure.

Group IV (MvitE): Male test group concomitantly administered with vitamin E daily

for the last two weeks of the exposure.

Group V (Fc): Female control group, without exposure to gasoline vapors.

Group VI (Ft): Female test group, exposed to gasoline vapors only.

Group VII (FvitA): Female test group concomitantly treated with vitamin A daily for the last two weeks of the exposure.

Group VIII (FvitE): Female test group concomitantly treated with vitamin E daily for the last two weeks of the exposure.

The rats were acclimatized in the experimental animal house for one week before the commencement of the experiment. The animals, housed in stainless steel cages under standard conditions (ambient temperature, 28.0 ± 2.0 °C and humidity, 46%, with a 12 hr light/dark cycle), were fed with the normal rat pellets. All the rats in both test and control groups were allowed free access to food and water *ad libitum*, throughout the experimental period. All the animal experiments were carried out in accordance with the guidelines of the Institution’s Animal Ethical Committee.

### Exposure to gasoline vapors

The animals in the test groups were wholly exposed to Premium Motor Spirit (PMS) blend unleaded gasoline (UG) vapors in a glass exposure chambers (1.5 m × 0.9 m × 2.1 m). The PMS blend liquid UG was obtained from Mobil refueling station, Marian Road, Calabar – Nigeria. The test animals were exposed to 17.8 cm^3^h^-1^m^-3^ (target concentration) of wholly vaporized PMS blend UG for 6 hr/day, 5 days/week, for 20 weeks. Exposure conditions were chosen to reproduce those used in our previous studies [4, 6, 7, 10]. Exposures were routinely conducted from 9.00 am to 3.00 pm on week days, including holidays to mimic workplace exposure. The chamber design, exposure generation system, and monitoring system were the same as those previously described [4, 6, 7, 10], with chamber concentrations of the UG determined daily. The average daily chamber concentrations of UG during exposure periods were 17.8 ± 2.6 cm^3^ (about 85 .4 percent of target concentration). At the end of the experimental period, the animals were sedated with chloroform and dissected for collection of blood specimen.

### Treatment of the rats with vitamins A and E

After eighteen weeks of pre-inhalation exposure to the gasoline vapors, the rats in groups III and VII were administered, once daily, with 400 IU/kg of vitamin A (retinol), while the rats in groups IV and VIII were administered, once daily, with 200 IU/kg of vitamin E (α-tocopherol) i.e., at normal prophylactic doses, concomitantly with exposure to gasoline vapors for the remaining two weeks. Administration of the vitamins was done by oral gavaging using intragastric syringe after solubilizing the vitamin with Goya Olive oil, as the vehicle.

### Collection and preparation of blood specimen for analyses

Blood samples were collected by cardiac puncture into plain screw-cap sample bottles. The blood samples collected were allowed to clot, and the serum extracted with Pasteur pipette after spinning with MSE model (England) table-top centrifuge at 2000 rpm for 5 minutes. The serum collected was used for biochemical analyses. All biochemical analyses were carried out within 24 hours of serum separation.

### Biochemical analyses

Biochemical analyses carried out included measurement of the concentration of alanine transaminase (ALT), aspartate transminase (AST), gamma-glutamyltransferase (GGT), alkaline phosphatase (ALP), and bilirubin in the serum. The measurements of the concentrations of these biochemical parameters were done by spectrophotometric determination of their absorbances, using analytical grade laboratory reagent kits. The laboratory reagent kits from Biosystems Laboratories (S. A. Costa Brava, Barcelonia, Spain) were used to assess the concentration of ALT, AST and ALP in the serum. While reagent kits from Randox Laboratories (United Kingdom) were used to assess the concentration of GGT, and bilirubin in the serum. All absorbance readings were taken with DREL3000 HACH model spectrophotometer.

### Statistical analyses

The results were analyzed by one-way analysis of variance (ANOVA) followed by Student’s t-test to evaluate the significance of the difference between the mean value of the measured parameters in the respective test and the control groups. A significant change was accepted at P < 0.05.

## Results

**Figure 1:**
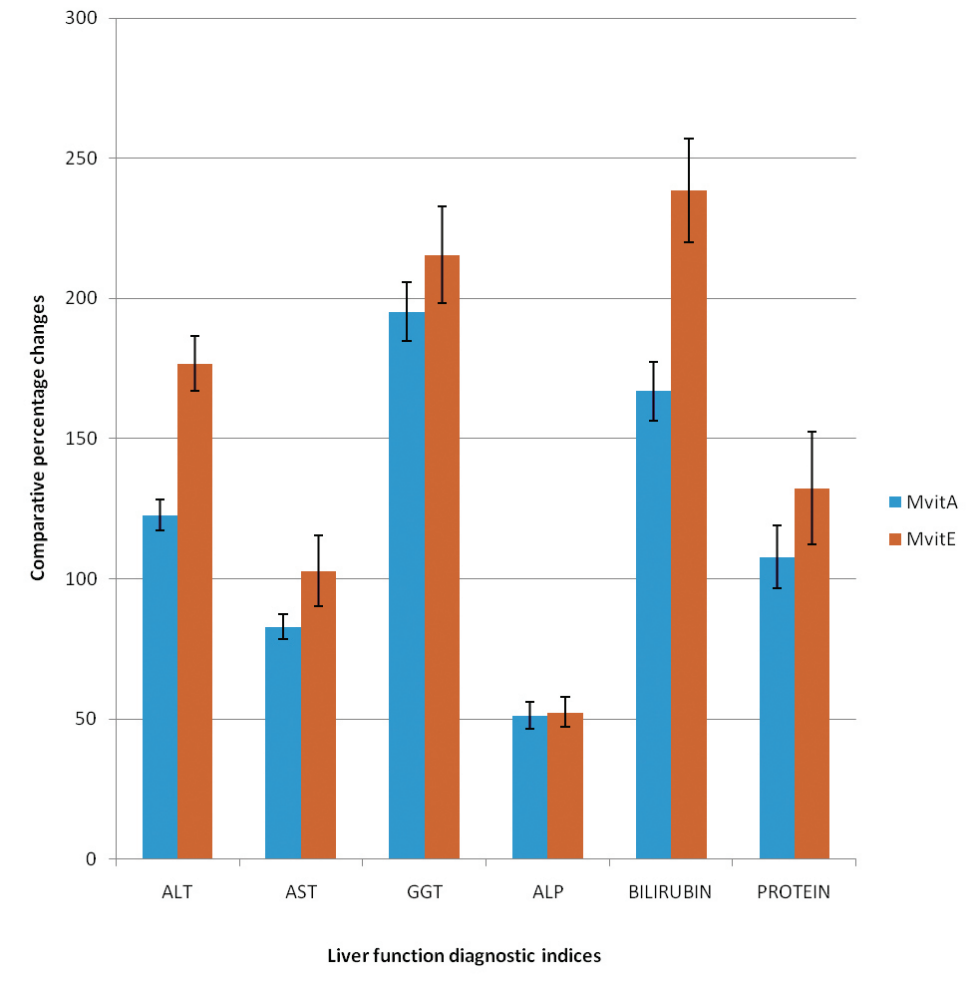
Comparative effect of gasoline vapors on some liver function diagnostic indices in male and female rats.

**Figure 2:**
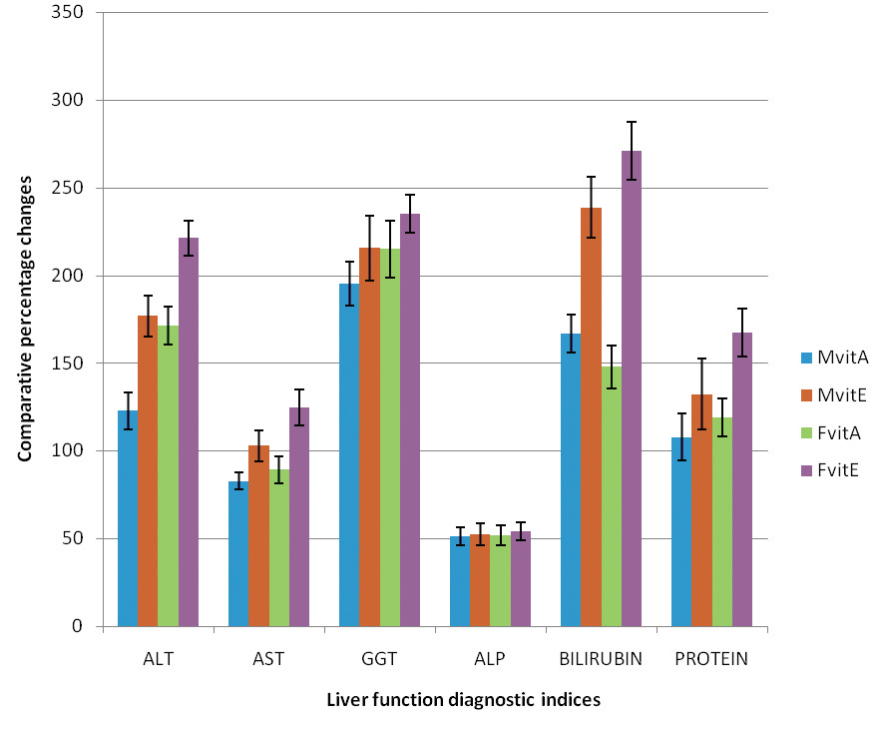
Comparative effect of vitamins A and E on comparative percentage changes in some liver function diagnostic indices in male and female rats exposed to gasoline vapors.

The results obtained from this present study are shown in tables 1 and 2, as well as in figures 1 and 2. From these results it was observed that the activities of ALT, AST, GGT and ALP obtained for both the male and female experimental test rats, exposed to gasoline vapor only, (i.e, Mt and Ft ), increased significantly (P < 0.05) when compared, respectively, with the activities obtained for the respective gender of rats in the control group ( i.e., Mc and Fc ) (Tables 1 and 2). These results showed that the comparative percentage increases in the activities of these enzymes obtained for female test rats were relatively higher compared, respectively, to the comparative percentage increase obtained for the male test rats (Fig. 1).

**Table 1:** Effect of vitamins A and E on the activities of some liver function diagnostic serum indices in male rats exposed to gasoline vapors

Group	ALT (U/L)	AST (U/L)	GGT (U/L)	ALP (U/L)	Bilirubin (µmol/L)	Total Protein(mg/dl)
I (Mc)	10.01 ± 2.14	20.67 ± 2.72	28.75 ± 2.30	269.65 ± 12.10	2.84 ± 0.60	6.75 ± 1.50
II (Mt)	25.05 ± 2.10*	40.25 ± 1.80*	88.20 ± 3.20*	408.36 ± 16.22*	8.06 ± 1.02*	2.85 ± 2.74*
III (MvitA)	11.25 ± 2.24^+,#^	22.02 ±1.60^+,#^	29.87 ± 2.34^+,#^	270.10 ± 20.16^+,#^	3.02 ± 2.10^+,#^	5.92 ± 2.60^+,#^
IV (MvitE)	9.05 ± 2.53**^,#^	19.85 ± 1.30**^,#^	27.96 ± 1.96**^,#^	267.98 ± 20.23**^,#^	2.38 ± 0.3 **^,#^	6.62 ± 2.56**^,#^

Values are presented as mean ± SEM, n = 6, *P < 0.05 compared with group I (control); ^+^P < 0.05 compared with group II; **P < 0.05 compared with group III; ^#^P > 0.05 compared with group I. Mc = male control; Mt = male test, exposed to gasoline vapors only; MvitA = male test treated with vitamin A; MvitE = male test treated with vitamin E.

**Table 2:** Effect of vitamins A and E on the activities of some liver function diagnostic serum indices in female rats exposed to gasoline vapors

Group	ALT (U/L)	AST (U/L)	GGT (U/L)	ALP (U/L)	Bilirubin (µmol/L)	Total Protein (mg/dl)
V (Fc)	9.35 ± 2.06	20.02 ± 2.46	27.73 ± 2.83	268.85 ± 13.12	2.56 ± 0.85	5.96 ± 1.82
VI (Ft)	27.18 ± 2.31*	41.01 ± 1.46*	90.01 ± 4.32*	411.63 ± 17.42*	8.38 ± 1.32*	2.55 ± 2.74*
VII (FvitA)	10.02 ± 2.24^+,#^	21.68 ± 2.06^+,#^	28.58 ± 2.83^+,#^	271.16 ± 18.15^+,#^	3.38 ± 2.10^+,#^	5.58 ± 2.60^+,#^
VIII (FvitE)	8.46 ± 2.53**^,#^	18.26 ± 1.23**^,#^	26.86 ± 2.06**^,#^	266.94 ± 20.52**^,#^	2.26 ± 0.3 **^,#^	6.82 ± 2.56**^,#^

Values are presented as mean ± SEM, n = 6, *P < 0.05 compared with group I (control); ^+^P < 0.05 compared with group II; **P < 0.05 compared with group III; ^#^P > 0.05 compared with group I. Fc = female control; Ft = female test, exposed to gasoline vapours only; FvitA = female test treated with vitamin A; FvitE = female test treated with vitamin E.

Furthermore, the results of this study showed that the activities of serum ALT, AST, GGT and ALP obtained for male and female test rats treated with retinol and α-tocopherol were significantly lower (P < 0.05 ) compared respectively to the activities of the enzymes obtained for male and female rats exposed to gasoline vapors only (Table 1). However, no significant difference was recorded in the activities of serum ALT, AST, GGT and ALP obtained for male and female test rats treated with retinol and α-tocopherol, when compared respectively with activities obtained for both male and female rats in the control group. Also, it was observed that the comparative percentage decreases in the activities of these enzymes in female test rats treated with retinol and α-tocopherol were significantly higher (P < 0.05 ) than the respective comparative percentage decreases obtained for male test rats under similar treatments; although the comparative percentage decreases in the activities these enzymes obtained for male and female test rats treated with α-tocopherol were observed to be significantly higher (P < 0.05 ) compared to the comparative percentage decreases in the activities these enzymes obtained for male and female test rats treated with retinol ( Fig. 2).

The results of this present study also reported that exposure to gasoline vapors caused a significant (P < 0.05 ), increase in the level of total serum bilirubin, and decrease in total serum protein in male and female rats compared to the control. As shown in figure 1, the comparative percentage increase in total serum bilirubin obtained for female rats exposed to gasoline vapors was significantly higher (P < 0.05 ) compared to the comparative percentage increase obtained for male rats under similar exposure condition, while no significant gender difference was recorded for total serum protein. Also, it was observed that the levels of total serum bilirubin in male and female rats treated with retinol and α-tocopherol were significantly lower (P < 0.05 ), compared respectively with the levels obtained for rats exposed to gasoline vapors only; whereas the levels of total serum protein obtained for male and female rats treated with the respective vitamins were significantly higher (P < 0.05 ), compared respectively with the levels obtained for rats exposed to gasoline vapors only (Tables 1 and 2). From these results, it was noted that the comparative percentage decreases in the levels total serum bilirubin, and increases in total serum protein obtained for male and female rats treated with α-tocopherol were significantly higher (P < 0.05 ) compared to the respective comparative percentage changes obtained for male and female test rats treated with retinol (Fig. 2).

Generally, the results of this study indicated that the percentage decreases in the activities of these serum enzymes and total serum bilirubin, as well as percentage increase in total serum protein levels, in both male and female test rats treated with α-tocopherol were significantly higher (P < 0.05 ) compared to the respective percentage changes obtained for both sexes of rats treated with retinol (Fig. 2). These results suggested that although retinol and α-tocopherol may enhance recovery from hepatotoxicity associated with exposure to gasoline vapors in male and female rats, the hepatoprotective potency of α-tocopherol tends to be higher than that of retinol. It is also clear from the results of this study that the beneficial effects of both retinol and α-tocopherol against gasoline vapors induced hepatotoxicity are relatively higher in females than the male rats.

## Discussion

Exposure to gasoline vapors is known to cause a wide spectrum of toxicological effects, which results in such biochemical dysfunctions that constitute serious health hazards to life. In animal model experiments, it has been reported that exposure to gasoline vapors produces various toxicity effects in many tissues. Proatherogenic changes in serum lipid profile and signs of hepatic oxidative stress [4-6], haematotoxicity [7, 8], reproductive toxicity [9] and nephrotoxicity [10], have earlier been reported to be associated with exposure to gasoline vapors in male and female rats. These reports gave a strong indication that gasoline vapors’ constituents participate in oxidative reactions associated with the generation of some reactive species (such as hydroxyl and superoxide radicals) which interact with membrane lipids of the respective tissues to produce lipid peroxides, similar to the condition reported for cadmium [23]. These reactive oxygen species, due to their high reactivity, provoke severe cellular alterations which can cause cell damage or death. The species attack important cell constituents such as proteins, lipids and nucleic acids. These lipid peroxides which accumulate in the tissues due to lipid peroxidation are known to be very harmful to cells and tissues [24].

The liver tissues have been reported to be one of the target organs of gasoline vapors toxicity [7]. The relationship between the hepatic oxidative damage and increase in the activities of serum ALT, AST, ALP and GGT has been well documented [6, 7, 19, 25]. Exposure to gasoline vapors stimulates the generation of cellular malondialdehyde (MDA), a product of lipid peroxidation [5], which affect the permeability barrier of the plasma membrane. The observations made from the result of this study strongly indicated that the hydrocarbons and other chemical constituents of the gasoline vapors are likely metabolized in the liver, among other tissues, to reactive species which interact with the tissues to cause lipid peroxidation, thereby exhibiting their toxic or hazardous effects. The increase in the activities of serum ALT, AST and ALP reported in this study may likely be caused by the reactive species induced membrane lipid peroxidation, resulting in the leakage of these cellular components into circulation [26]. Hence, the increased serum activities of these liver enzymes reported in this present study is likely to be caused by the accumulation of the gasoline vapors’ constituents and their reactive metabolites in hepatic tissues which enhances formation of lipid peroxidation.

In this present study, a significant increase in total serum bilirubin, and decrease in total serum protein levels is also reported to be associated with exposure to gasoline vapors in male and female rats. It has been reported that increase in plasma or serum bilirubin level may be a useful indication of either or all of these conditions; excessive production of bilirubin as in haemolytic anaemia, reduced hepatic uptake as in the liver subjected to potential damage from an enormous array of chemical agents, impaired bilirubin conjugation, decreased hepatocellular excretion, and impaired intrahepatic and extrahepatic bile flow [27, 28]. Although the specific mechanism(s) responsible for the reported increase in the total serum bilirubin level, is not very clear, the result of the enzyme studies supported the indication that the gasoline vapors’ constituents might have interacted with the liver tissues to raise the total serum bilirubin level through one or more of the mechanisms earlier outlined by Crawford [28]. This observation supports our earlier and present reports that frequent exposure to gasoline vapors caused a significant increase in the activities of serum ALT, AST, ALP, GGT and total bilirubin, as well as relative liver weight, hence hepatotoxicity, in male and female rats. The reduction in total serum protein reported in this study is also pointer to the possibility of a compromise in the liver functions following exposure to gasoline vapors. The observations made from the results of this study indicated that the hepatotoxic effects associated with exposure to gasoline vapors may results in diverse clinical complications.

From results of this present study, retinol and α-tocopherol were observed to produce a significant reduction in the increased serum ALT, AST, ALP, GGT activities and bilirubin level, as well as a significant increase in the reduced total serum protein level reported to be caused by exposure to gasoline vapors in male and female rats. This observation indicated that retinol and α-tocopherol possess hepatoprotective property against gasoline vapors toxicity in male and female rats. The results of this study therefore strongly support the antioxidative and protective roles of vitamins A and E reported in the literature [12, 16, 17]. Prevention of reactive metabolites formation or rapid scavenging of the generated reactive species by the antioxidants may be useful in preventing the toxicity effects of different reactive metabolites. For example, Peshlon and Hesse [29], Maellaro et al [30] and Sheweita et al [31] reported that antioxidants have proved to be effective in protecting the liver against carbon tetrachloride induced hepatotoxicity. With the antioxidative and protective roles of these vitamins, the toxic effects of the various reactive metabolites responsible for the hepatotoxicity observed to be associated with exposure to gasoline vapors, are assumed to have been reversed or prevented. The results of this also agree with the report of Bashandy and Alhazza [19], that β-carotene protects the liver against cadmium toxicity in rats; this report recorded that β-carotene reduced cadmium-induced elevation of ALT, AST and ALP in rats. Beta-carotene, the precursor of vitamin A, has been reported to suppress lipid peroxidation elevation in rats by quenching the activities of free radicals and prevent them from inducing oxidative stress [32, 33]. According to McNulty et al [34], carotenoids quench singlet oxygen primarily by physical mechanism, in which excess energy of the singlet oxygen is transferred to carotenoids and then they relaxes into ground state, as a result carotenoids offer to protect against further oxygen radical and lipid peroxidation.

With the gasoline vapors-induced toxicity protective role of retinol and α-tocopherol reported in this study, it evidently clear that retinol and α-tocopherol serve as antioxidant, preventing or reversing the toxic effects of the various reactive metabolites responsible for the hepatotoxicity observed to be associated with exposure to gasoline vapors in rats. Although the result of this present work also indicated that the beneficial effects of retinol and α-tocopherol against gasoline vapors induced hepatotoxicity was relatively higher in females than the male rats, the hepatoprotective potency of α-tocopherol was observed to be relatively higher than that of retinol in both male and female rats. The observed higher hepatoprotective potency reported in this study for α-tocopherol over retinol agrees with the report of Machlin and Bendich [35] that α-tocopherol is the major lipid soluble antioxidant vitamin protecting membranes and lipoproteins from injury by free radicals. These vitamins are believed to improve the activities of antioxidative enzymes following oxidative stress; and being lipophilic molecules, they are likely to exert their action in such hydrophobic environment as the lipid core of membranes, thereby protecting the tissues from damages. Moreover, the specific mechanism(s) responsible for the observed relative sex-dependent hepatoprotective beneficial effect of the vitamins is a subject for further studies.

In conclusion, the results of this study showed that although retinol and α-tocopherol may be used to enhance the protection of the liver tissues against hepatotoxicity associated with exposure to gasoline vapors, the hepatoprotective potency of α-tocopherol is higher than that of retinol in both male and female rats. Also the beneficial effect of these vitamins against gasoline vapors induced hepatotoxicity tends to be sex-dependent, with females responding higher than male rats.
